# Comment on “Neuromuscular Impairment at Different Stages of Human Sarcopenia” by Sarto et al.—The Authors' Reply

**DOI:** 10.1002/jcsm.13766

**Published:** 2025-03-05

**Authors:** Fabio Sarto, Evgeniia Motanova, Marco V. Narici

**Affiliations:** ^1^ Department of Biomedical Sciences University of Padova Padova Italy; ^2^ CIR‐MYO Myology Centre University of Padova Padova Italy

We read with interest the comment by Jones et al. [1] regarding our recent article “Neuromuscular impairment at different stages of human sarcopenia” [2]. We are grateful for their thoughtful engagement with our study and their recognition of its contribution to understanding the effects of ageing and sarcopenia on the human neuromuscular system. Their relevant observations offer an excellent opportunity to further discuss the challenges of neuromuscular junction (NMJ) evaluation in human research.

We fully acknowledge the importance of direct morphological assessment to determine NMJ structural integrity and potential denervation. Indeed, despite the technical and logistical challenges of obtaining NMJ‐positive muscle samples in humans, we are currently conducting research projects that incorporate morphological studies of NMJs, both in the context of ageing and under conditions of muscle disuse (manuscripts currently in preparation). These ongoing efforts align with our commitment to providing a comprehensive understanding of NMJ remodelling in different scenarios.

That said, we believe that the findings presented in our study remain novel, relevant and informative, even in the absence of direct NMJ morphological data. Our conclusions were based on a rigorous and comprehensive combination of methodologies, including gold‐standard electrophysiological analyses [3], well‐established circulating biomarkers of neuromuscular degeneration [4, 5] and robust molecular analyses of muscle tissue [6]. Together, these approaches provided converging evidence of age‐related alterations of human neuromuscular system, which, while indirectly, may reflect significant processes associated with NMJ instability and altered innervation profile.

We thank Jones et al. for their valuable comments and welcome the ongoing dialogue on this important topic.

## Conflicts of Interest

The authors declare no conflicts of interest.
